# Angelman Syndrome With Papillary Urothelial Carcinoma: An Unusual Initial Presentation and Two Recurrences

**DOI:** 10.7759/cureus.93903

**Published:** 2025-10-05

**Authors:** Faisal Altwijri, Osama Almubadel, Naif Alateeq, Faisal Alsaleh, Mohammad Jouma, Abdelmoniem Elgaili El-Tiraifi

**Affiliations:** 1 Urology, King Khalid University Hospital, Riyadh, SAU; 2 Medicine, King Saud University, Riyadh, SAU; 3 Medicine, King Khalid University Hospital, Riyadh, SAU

**Keywords:** angelman syndrome, bladder cancer, neurodevelopmental disorders, rare case, ube3a gene, urothelial carcinoma

## Abstract

Bladder cancer, particularly urothelial carcinoma, is the most common malignancy of the urinary tract and is associated with several risk factors, including smoking, advanced age, and exposure to environmental toxins. Angelman syndrome is a rare neurodevelopmental disorder caused by a defect in the UBE3A gene, leading to characteristic neurological and behavioral manifestations. Although no clear association exists between these two conditions, we report a case of a 36-year-old man with Angelman syndrome who developed urothelial carcinoma of the urinary bladder despite the absence of identifiable risk factors or a family history of urothelial cancer, followed by two subsequent recurrences.

## Introduction

Urothelial carcinoma is the most common type of bladder cancer, accounting for approximately 90% of all cases. This cancer arises from the urothelial cells, which form the inner lining of the bladder. Risk factors for bladder cancer include smoking, occupational exposure to chemicals, and chronic bladder inflammation [1,2]. Urothelial carcinoma rarely occurs in the absence of these risk factors, particularly in young patients, as seen in our case. Angelman syndrome is a rare neurodevelopmental genetic disorder caused by abnormalities in the UBE3A gene located on chromosome 15q11-q13. The UBE3A gene normally acts as a cellular “quality control” system, tagging unwanted proteins for breakdown to maintain healthy nerve cell function. It affects approximately 1 in 15,000 individuals. Patients with Angelman syndrome typically present with intellectual disability, seizures, motor dysfunction, and severe speech impairment [3]. The UBE3A gene plays a key role in protein degradation and neuronal function [4]. Recent evidence suggests a potential association between Angelman syndrome and bladder cancer, as both conditions involve abnormalities on chromosome 15q. This overlap raises the possibility of a shared genetic mechanism contributing to disease development. Previous studies have reported deletions and loss of heterozygosity in this chromosomal region in cases of bladder cancer among young patients [4,5]. In our report, we describe a rare case of a 36-year-old man with Angelman syndrome who developed low-grade, non-invasive papillary urothelial carcinoma of the bladder, followed by two subsequent recurrences. This case adds to the growing interest in exploring possible associations between Angelman syndrome and malignancies such as bladder cancer. By documenting this case, we aim to highlight the clinical relevance of these findings and encourage further research into potential genetic links between neurodevelopmental syndromes and cancer.

## Case presentation

**Figure 1:**
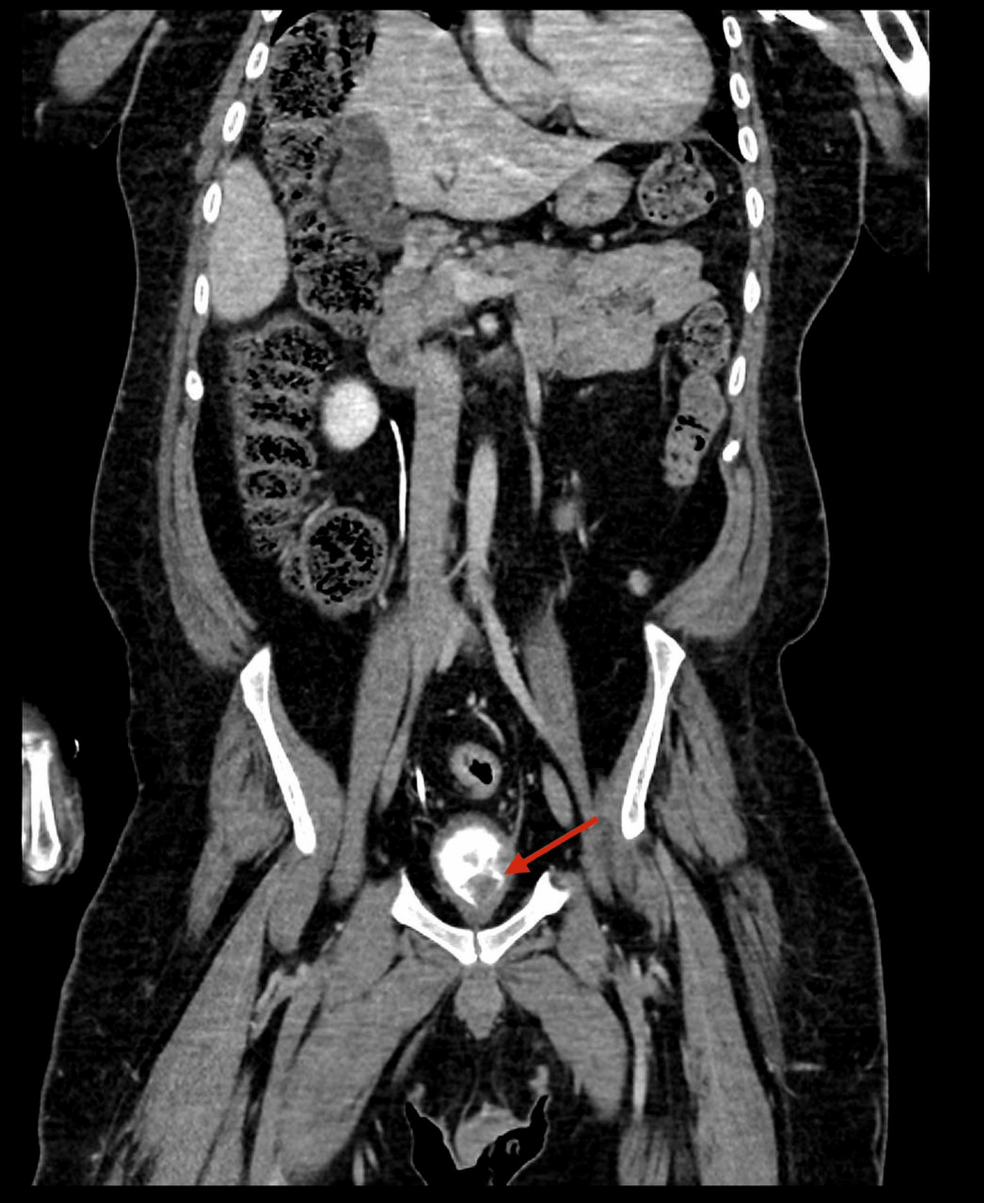
CT urography (coronal view) showing a bladder tumor (arrow) on the left lateral wall. The mass enhances irregularly and protrudes into the bladder cavity, creating a filling defect and distorting the bladder outline. Abnormal finding: irregular intravesical mass. Normal findings: no hydronephrosis, no upper tract involvement, and no spread outside the bladder.

**Figure 2:**
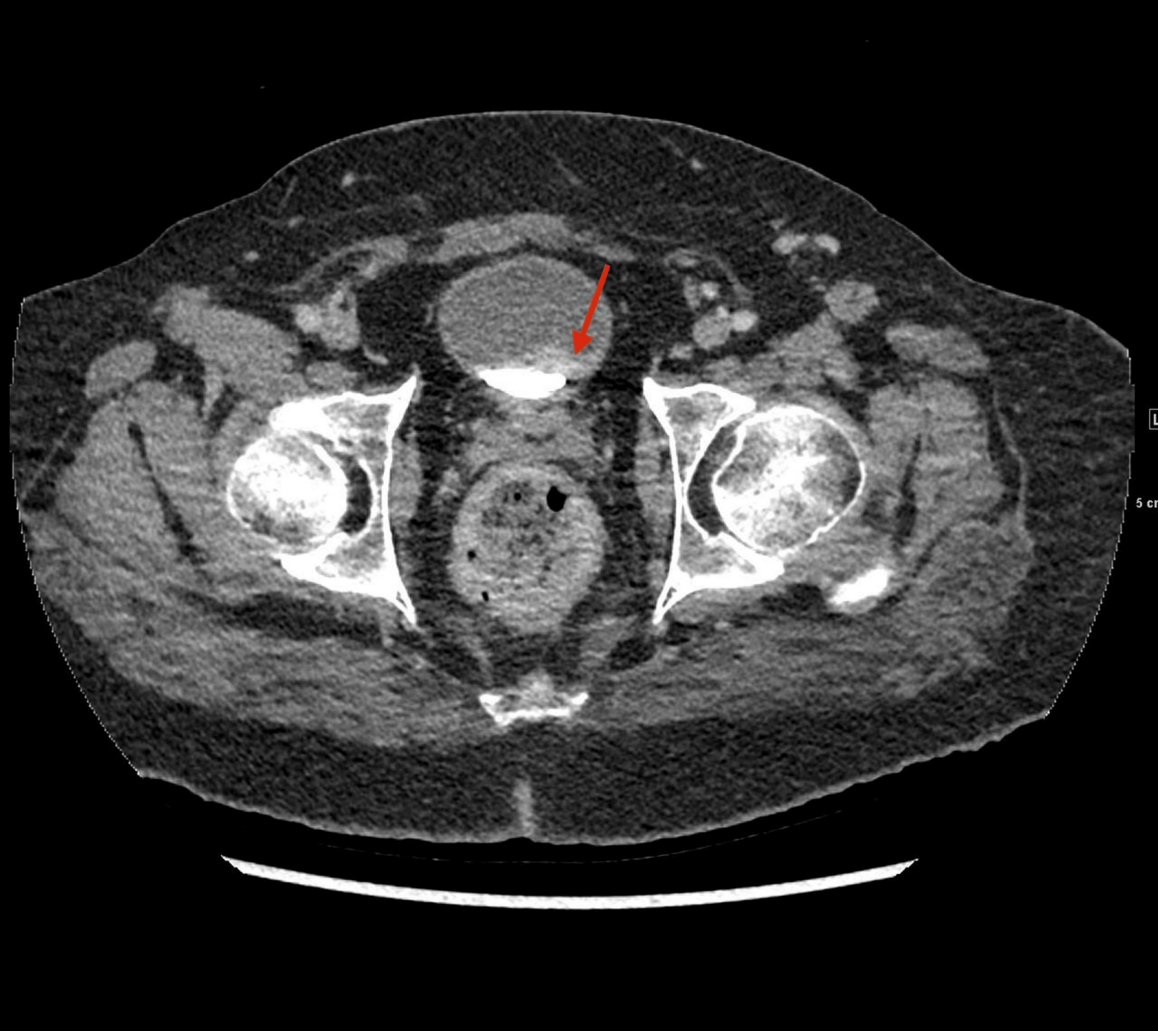
CT urography (axial view) showing a heterogeneous soft-tissue mass (arrow) arising from the left lateral bladder wall. The lesion projects into the bladder lumen as a filling defect with an irregular, enhancing contour, consistent with a bladder tumor. Abnormal finding: irregular intravesical mass. Normal findings: surrounding pelvic structures (prostate and pelvic bones) are intact with no spread outside the bladder.

**Figure 3:**
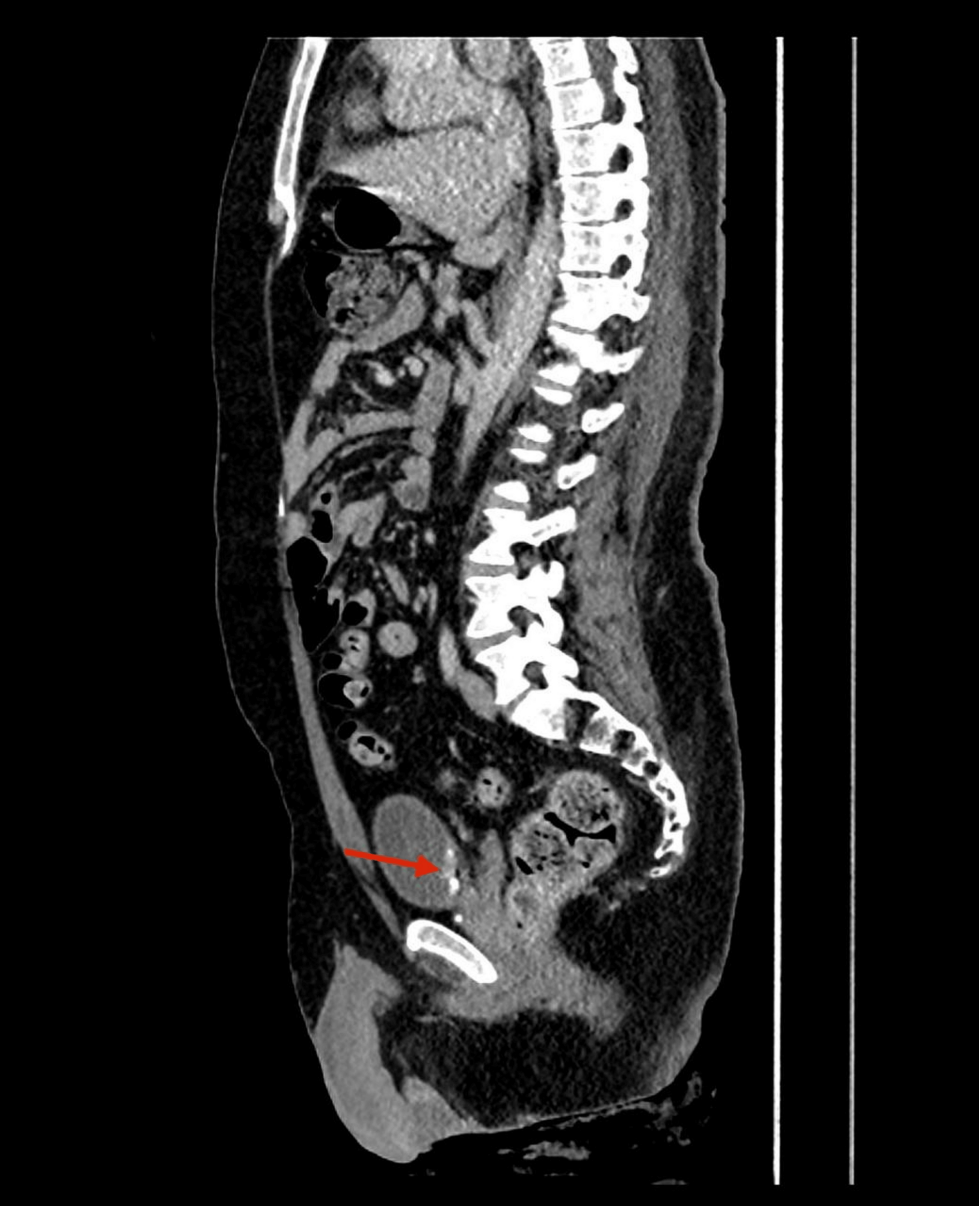
CT urography (sagittal view) showing a heterogeneous enhancing mass (arrow) arising from the left bladder wall. The tumor projects into the bladder lumen as a filling defect with irregular contours. The sagittal plane highlights the depth of bladder wall involvement. Abnormal finding: irregular intravesical mass with wall distortion. Normal findings: no evidence of spread outside the bladder, with surrounding pelvic structures preserved.

**Figure 4:**
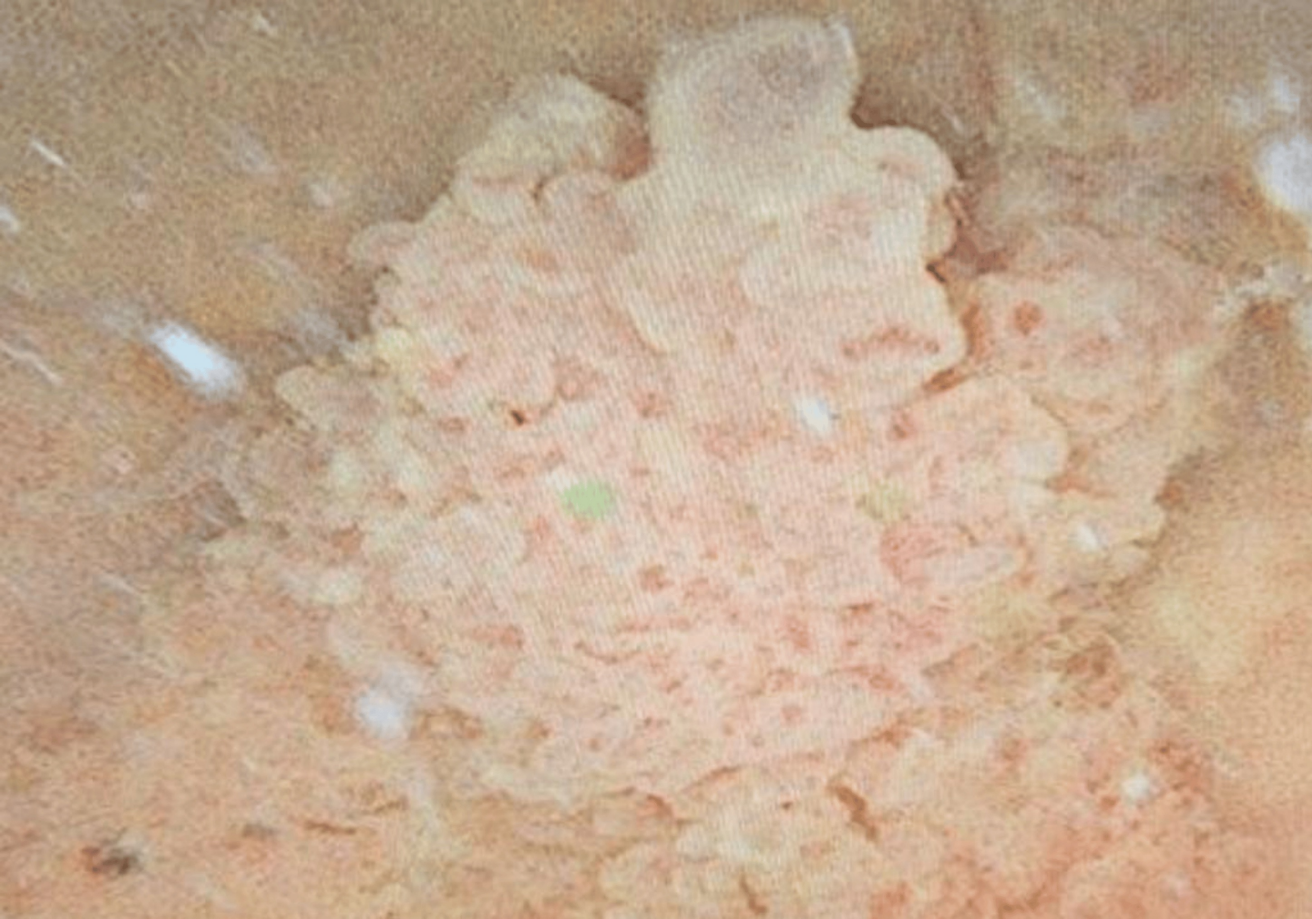
Intraoperative cystoscopy showing a large papillary, exophytic mass (arrow) arising from the left bladder wall. The lesion has an irregular surface with frond-like projections and protrudes into the bladder lumen, consistent with urothelial carcinoma. Abnormal finding: papillary bladder tumor with intravesical growth. Normal findings: remainder of bladder mucosa not involved in this view.

**Figure 5:**
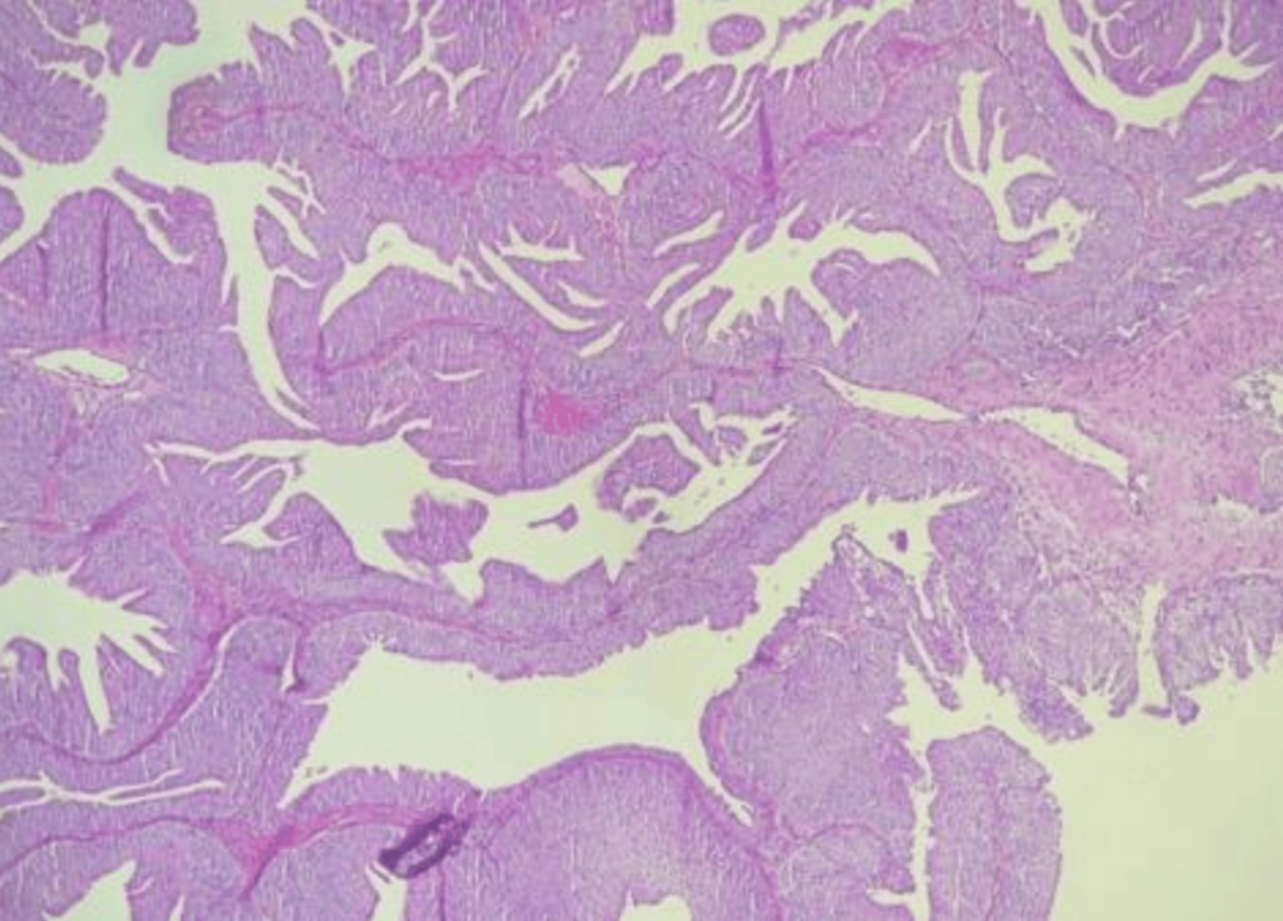
Microscopic histology revealing low-grade, non-invasive papillary urothelial carcinoma. Histopathology showing delicate papillary fronds lined by urothelium with mild nuclear atypia and preserved polarity. Abnormal finding: nuclear atypia without loss of polarity. Normal findings: no invasion of the lamina propria or muscularis propria. Diagnosis: low-grade, non-invasive papillary urothelial carcinoma.

A 36-year-old man with a known history of Angelman syndrome, characterized by neurological and cognitive manifestations such as epilepsy and intellectual disability, also had diabetes mellitus, hypertension, and a prior hemorrhagic stroke. He had an unremarkable family history and presented with a one-week history of asymptomatic gross hematuria. On examination, the patient was stable, afebrile, and not in distress. Abdominal and external genital examinations were unremarkable. Vital signs were within normal limits: axillary temperature 36.5 °C, heart rate 84 beats per minute, respiratory rate 19 breaths per minute, blood pressure 125/76 mmHg, and mean arterial pressure 92 mmHg (Table 1). The complete blood count (CBC) and routine chemistry were within normal limits (Table 2). Urinalysis was positive for red blood cells, protein, and white blood cells. CT urography revealed a left bladder wall mass (Figures 1-3). The patient underwent cystoscopy under general anesthesia due to pain intolerance, which revealed a large papillary mass on the left bladder wall (Figure 4). Transurethral resection of the bladder tumor (TURBT) was performed, followed by a single dose of mitomycin without complications. Histopathological examination confirmed low-grade, non-invasive papillary urothelial carcinoma (Figure 5). The patient was scheduled for follow-up cystoscopy after three months. If negative, additional cystoscopies were planned at nine months and then annually for five years. At the first three-month follow-up, the patient remained stable, with unremarkable laboratory results. A single small (<1 cm) papillary lesion was identified, resected via TURBT, and confirmed as low-grade, non-invasive papillary urothelial carcinoma without muscularis propria tissue identified. A second follow-up cystoscopy was performed three months later. The patient remained clinically stable with normal laboratory findings. The urethra appeared normal, the prostate was non-obstructing, and both ureteric orifices showed clear bilateral urine efflux. The previous resection site was free of recurrence; however, a new small (<1 cm) lesion was found on the right lateral bladder wall. It was resected via TURBT and again confirmed histologically as low-grade, non-invasive papillary urothelial carcinoma without identification of muscularis propria tissue. Intravesical mitomycin was administered 90 minutes postoperatively, followed by an induction course of weekly doses for five consecutive weeks.

**Table 1:** Vital signs at presentation. The patient’s vital signs were within normal limits, indicating clinical stability at presentation.

Parameter	Value	Unit
Axillary temperature	36.5	°C
Heart rate	84	Beats per minute
Respiratory rate	19	Breaths per minute
Blood pressure	125/76	mmHg
Mean arterial pressure	92	mmHg

**Table 2:** Complete blood count (CBC) and routine chemistry results. Laboratory investigations were largely unremarkable. Notable findings included elevated total and direct bilirubin, low alkaline phosphatase, and mildly decreased albumin. AST: aspartate transaminase, ALT: alanine transaminase, ALP: alkaline phosphatase.

Parameter	Result(s)/range	Reference range*
CBC		
WBC	8.2 × 10⁹/L	4-11 × 10⁹/L
Hemoglobin	14.6 g/dL	13-17 g/dL
Hematocrit	45.4%	40%-50%
Platelets	279 × 10⁹/L	150-400 × 10⁹/L
Neutrophils	53%	40%-70%
Lymphocytes	36%	20%-45%
Monocytes	6%	2%-10%
Eosinophils	3%	1%-6%
Basophils	1%	0%-2%
Chemistry		
AST	10.0 U/L	<40 U/L
ALT	11.0 U/L	<41 U/L
ALP	30.0 U/L	40-129 U/L
Direct bilirubin	3.70 mg/dL	<0.5 mg/dL
Total bilirubin	5.90 mg/dL	0.1-1.2 mg/dL
Sodium	137.0-142.0 mmol/L	135-145 mmol/L
Potassium	3.90-5.40 mmol/L	3.5-5.1 mmol/L
Chloride	99.87-107.0 mmol/L	98-106 mmol/L
CO₂ (bicarbonate)	23.40-29.71 mmol/L	22-29 mmol/L
Calcium	2.19-2.46 mmol/L	2.15-2.55 mmol/L
Total protein	63.0-78.0 g/L	60-80 g/L
Albumin	31.0-39.0 g/L	35-50 g/L
Uric acid	239.0-330.0 µmol/L	200-430 µmol/L
Creatinine	65.0-90.0 µmol/L	60-110 µmol/L
Urea	3.10-7.90 mmol/L	2.5-7.1 mmol/L

## Discussion

Our report presents a rare case of a 36-year-old man with Angelman syndrome who developed urothelial carcinoma of the urinary bladder despite the absence of known risk factors, including smoking, occupational or environmental toxin exposure, or a family history of genitourinary malignancy, and subsequently experienced two recurrences. Urothelial carcinoma is a tumor that originates from the urothelial cells lining the urinary tract [1]. Urothelial carcinoma is the most common type of bladder cancer, accounting for about 90% of all cases [1]. In 2022, there were approximately 614,298 new cases of bladder cancer globally, representing a 7.1% increase compared to 2020. Bladder cancer is more common in males than in females, and there are several risk factors for this type of cancer, including smoking, chemical exposure, and chronic bladder inflammation [2,6]. Angelman syndrome is a rare neurodevelopmental disorder affecting approximately 1 in 15,000 individuals. Patients with Angelman syndrome typically present with intellectual disability, seizures, motor dysfunction, and speech impairment [3]. Angelman syndrome is caused by an abnormality in the maternally expressed UBE3A gene on chromosome 15q11-q13, which encodes an E3 ubiquitin ligase essential for neurological function. There are four molecular mechanisms underlying Angelman syndrome: maternal deletion of 15q11-q13, UBE3A mutations, paternal uniparental disomy, and imprinting defects. All of these mechanisms result in the loss of maternal UBE3A expression [4]. The UBE3A gene does not have a direct link to cancer; however, it is involved in many pathways related to cancer biology, including protein degradation. This highlights the possibility of finding an association between Angelman syndrome and an increased risk of cancer [5]. Previous studies have reported frequent deletions and loss of heterozygosity (LOH) on chromosome 15q in patients with transitional cell carcinoma of the bladder. This region partially overlaps with 15q11-q13, which is implicated in Angelman syndrome due to deletions or mutations in the maternal copy of the UBE3A gene. While the deletions observed in bladder cancer may involve tumor suppressor genes contributing to carcinogenesis, and those in Angelman syndrome affect neurodevelopment, a direct causal relationship between the two conditions has not been established [4,7]. Nonetheless, this chromosomal overlap raises the possibility of shared genetic vulnerabilities or molecular mechanisms, highlighting the need for further research. In addition, a previous case described a 22-year-old woman with Angelman syndrome who presented with a history of premenstrual cyclic nausea, vomiting, and abdominal pain, with multiple prior hospitalizations for these symptoms. Incidentally, a pelvic ultrasound revealed a 7 mm echogenic focus along the posterior bladder wall. Further evaluation with cystourethroscopy under anesthesia identified a 1.5 cm papillary lesion on the right bladder sidewall. She was managed with complete transurethral resection of the bladder tumor followed by surveillance cystoscopy under anesthesia every three months [8]. Diagnosis of urothelial carcinoma is reached by cystoscopy and transurethral resection of bladder tumor, which is sent to pathology [9,10]. Management of urinary bladder urothelial carcinoma is dependent on grading: if the patients has non-muscle-invasive bladder cancer, such as the patient reported, the management will be by transurethral resection of bladder tumor and then intravesical therapy with an immediate postoperative single dose of intravesical chemotherapy such as mitomycin C to reduce the risk of recurrence and immunotherapy, and then regular cystoscopies will be done for the patient for early detection of recurrence [11]. This case, along with ours, highlights the importance of further research to explore the possible association between Angelman syndrome and bladder cancer, as well as other types of malignancies.

## Conclusions

This case report presents an extraordinary and relatively unusual case of urothelial carcinoma in a 36-year-old man with Angelman syndrome, a genetic disorder characterized by developmental delays, who subsequently experienced two recurrences. Thus far, there is no definitive association between Angelman syndrome and urothelial carcinoma, which makes this discovery essential for identifying a possible malignancy danger. The investigation of potential correlation between Angelman syndrome and cancer should not be dismissed because the patient has not had the risk factors such as smoking, toxins, or family history. Management, such as TURBT and surveillance cystoscopy, suggests the need for anticipatory measures in such cases. Therefore, cystoscopy screening in patients with Angelman syndrome may be considered a topic for further study, given the potential role of the UBE3A gene in malignancy regulation. This case highlights the importance of conducting further research on the underlying relationship to establish an ideal diagnosis and management in the early stages of such rare and complex diseases, thereby enabling patients to have a better prognosis.
